# Effective Dose Radon 222 of the Tap Water in Children and Adults People; Minab City, Iran

**DOI:** 10.5539/gjhs.v8n4p234

**Published:** 2015-08-31

**Authors:** Yadolah Fakhri, Morteza Kargosha, Ghazaleh Langarizadeh, Yahya Zandsalimi, Leila Rasouli Amirhajeloo, Mahboobeh Moradi, Bigard Moradi, Maryam Mirzaei

**Affiliations:** 1 Social Determinants in Health Promotion Research Center, Hormozgan University of Medical Sciences, Bandar Abbas, Iran; 2 Food and Drugs Research Center, Bam University of Medical Sciences, Bam, Iran; 3 Environmental Health Research Center, Kurdistan University of Medical Sciences, Sanandaj, Iran; 4 Department of Environmental Health Engineering, School of Public Health, Qom University of Medical Sciences, Qom, Iran; 5 Department of Environmental Health Engineering, School of Public Health, Shahid Beheshti University of Medical Sciences, Tehran, Iran; 6 Department of Health Public, Kermanshah University of Medical Sciences, Kermanshah, Iran; 7 Jahrom University of Medical Sciences, Jahrom, Iran

**Keywords:** Radon 222, Effective dose, tap water, child and adults humans

## Abstract

^222^Rn is a radioactive, odorless, and colorless element which has a half-life of 3.83 days. One of ^222^Rn main resources are Groundwater (wells, springs, etc.). Hence, the use of groundwater with high concentration of ^222^Rn can increase the risk of lung and stomach cancers. Concentration of ^222^Rn in tap water of Minab city in two temperatures 5 and 15 ºC was measured by radon meter model RTM1668-2. The effective dose was calculated by equations proposed by UNSCEAR. Geometric mean concentration of ^222^Rn in drinking water was found to be 0.78±0.06 and 0.46±0.04 Bq/l at 5 and 15 °C (p value<0.05), respectively. The effective doses were 0.006 and 0.003 mSv/y for adults, and 0.011 and 0.007 mSv/y for the children, respectively (p value<0.05). Besides, the effective dose for adult through inhaling ^222^Rn at 5 and 15 °C were estimated 0.0021 and 0.0012mSv/y, respectively. Geometric mean concentration in ^222^Rn drinking water and effective dose received from drinking water and inhalation of ^222^Rn is lower than WHO and EPA standard limits. Increasing temperature of drinking water will decrease the effective dose received. Annual Effective dose received from inhalation and consumption of ^222^Rn in drinking water in children is more than adults.

## 1. Introduction

Radon 222 (^222^Rn) is produced as a result of decay of Radium 226 (^226^Ra) in Uranium 235 (^235^U) chain. This element is radioactive, odorless, colorless, and water soluble and has a half-life of 3.83 days (Al-Khateeb, Al-Qudah, Alzoubi, Alqadi, & Aljarrah, 2012; Ju, Ryu, & Jang, 2012). Several studies indicate that ^222^Rn indoor air concentration have a significant relationship with lung cancer (Torres-Durán, Barros-Dios, Fernández, & Ruano-Ravina, 2014). Indoor air death rate from ^222^Rn has been announced approximately 21,000 people a year, 10 times more than air pollution deaths (Environmental Protection Agency, 2010). Studies have shown that ^222^Rn, received the annual effective dose 1.3mSv/y due to natural exposure (2.4 mSv/y) to dedicate (Over 50%) (Magill & Galy, 2005). United Nations Scientific Committee on the effects of atomic radiation (UNSCEAR) has expressed exposure standard effective dose received from natural radioactive 2.5 mSv/y, which is 1 mSv/y related to ^222^Rn (Mehra and Bala, 2013; Radiation, 2000). ^222^Rn in drinking water can enter the internal organs such as the stomach and cause cancer (Somlai, Tokonami, Ishikaw, Vancsur, Gáspár, 2007; Auvinen, Salonen, Pekkanen, Pukkala, & Ilus, 2005; Alizadeh, Mahvi, & Fakhri, 2014). Also ^222^Rn inhalation can cause damage to DNA lung cells and leads to lung cancer in the population (Todorovic, Nikolov, Forkapic, Bikit, & Mrdja, 2012; Motesaddi, Fakhri, Alizadeh, Mohseni, & Jafarzadeh, 2014). European Commission and the World Health Organization has proposed concentration of ^222^Rn in the drinking water, 100 Bq/l as the standard limit (WHO, 2006). EPA 11 Bq/l, has been suggested as the maximum concentration Level (MCL) of ^222^Rn in drinking water (Environmental Protection Agency, may 2012). Many studies have shown that groundwater resources rather than surface water resources have much higher concentration of radioactive materials such as ^222^Rn (Amin, 2013; Rožmarić, Rogi, Benedik, & Štrok, 2012). The total indicative dose (TID) induced by radioactive substances (^3^H, ^40^K, ^222^Rn) as well as those produced through ^222^Rn decayed in drinking water is reported to be 0.1 mSv/y by WHO and the European committee (Somlai at al., 2007; Todorovic at al., 2012; WHO, 2004). Due to the exit of ^222^Rn during water transfer in distribution network, water transfer from one container to another, storing and boiling water, determining the standardized effective dose induced by ^222^Rn is difficult (Ishikawa, Tokonami, Yoshinaga, & Narazaki, 2005). Hence, the effective standard level dose 0.1 mSv/y is used for the analysis. In the present study, the effective dose of ^222^Rn received by children and adult age groups in Minab drinking water was calculated.

## 2. Materials and Methods

### 2.1 Study Area

**Figure 1:**
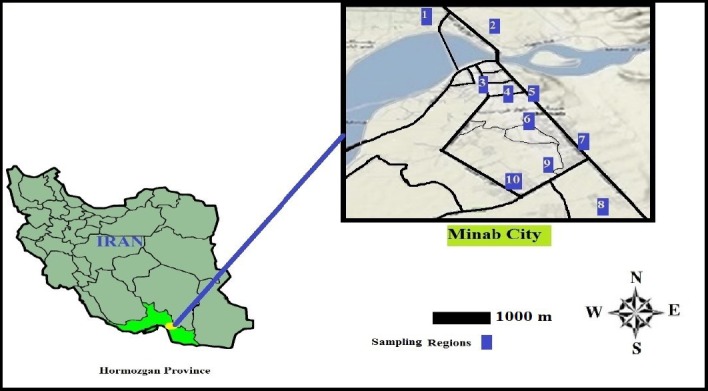
sampling regions of Minab in the East province of Hormozgan, Iran (2)

Minab city with a population of approximately 90 thousand people is located in geographic coordinates 27º06’40N and 57º05’52, at an elevation of 45 meters above sea level (Figure 1). The city is located in a hot and humid region and the water consumption per capital was high. The only water resources in this town are three deep wells (Groundwater source), the water of which is pumped out and distributed with no purification process.

### 2.2 Sample Collection

Since the retention time of water in the distribution network is effective on the concentration of ^222^Rn (Ishikawa, Tokonami, Yoshinaga, & Narazaki, 2005), thus, the sampled locations were determined from the beginning to the end of the distribution network. For 4 consecutive months, the sampling was done in 10 regions of the town. Meanwhile, 25 samples were selected from each region. During each stage of time, a total of 250 samples, each containing 2l of city tap water were obtained from 10 regions Sampling was conducted according to the proposed method (EPA).

### 2.3 Measurement Concentration of ^222^Rn

**Figure. 2:**
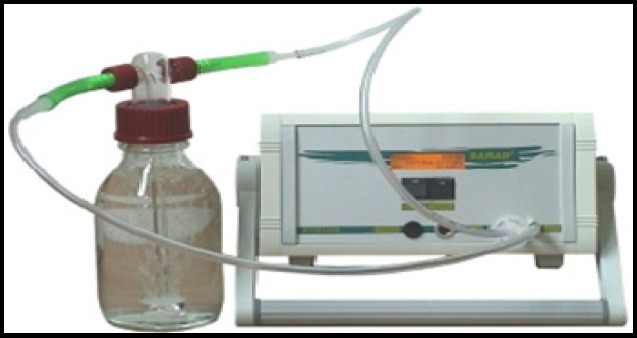
Measurement water ^222^Rn levels by RTM 1688-2 device, manufactured by Sarad corporation in Germany.

Measurement of radioactive substances in water, soil and air are done in various ways, such as alpha spectrophotometry, inductively coupled plasma/mass spectrophotometer, gamma spectrophotometry and liquid Scintillation (Rožmarić, Rogi, Benedik, & Štrok, 2012). Recently, many studies measure the concentration of ^222^Rn portable devices, such as RAD7 RTM (Mehra and Bala, 2013; Ju, Ryu, Jang, Dong, & Chung, 2012; Todorovic, Jovana Nikolov, Sofija Forkapic, Istvan Bikit, & Dusan Mrdja, 2012; Lee & Kim, 2006). Hence, in this study a model of portable alpha spectrophotometry RTM1688-2 was used to measure ^222^Rn in drinking water. To determine the effect of water temperature on the diffusion rate ^222^Rn of water, measurements was done at 5 and 15ºC temperatures. According to measurement of 300 mL, after the sample size reached the intended temperature, the device was placed in a closed cycle (Figure 2). The time for balance between concentration of ^222^Rn and its decay products (daughters ^222^Rn) is 4 hour approximately (Ju, Ryu, & Jang, 2012; Mehra & Bala, 2013; Lee & Kim, 2006). Hence, the 4 hour mean concentration of ^222^Rn (Bq/l) and the initial temperature (ºC) was recorded.

### 2.4 Calculation of effective dose (Ingestion)

To determine ^222^Rn annual effective dose received in the stomach from water consumption, Equation 1 was used (Sarad, 2009). In this equation E: the annual effective dose received by mSv/y, K: Coefficient conversion concentration of ^222^Rn to effective dose According to mSv/Bq, KM: Annual water consumed l/y, C: the concentration of ^222^Rn depending on Bq/l and T: the period of water consumption in the study, here was 365 days (Somlai, Tokonami, Ishikawa, Vancsura, & Gáspár, 2007).


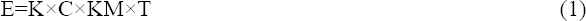


Conversion factor for adults and children were 1×10^-8^ Sv/Bq and 2×10^-8^ Sv/Bq, respectively (Amin, 2013; WHO, 2004). KM is daily consumption which is considered 2 l/d.

### 2.5 Calculation of the Effective Dose (Inhalation)

In order to estimate the effective dose received annually through inhaling ^222^Rn of underground water, the conversion coefficient of 2.8 µSv.lit/Bq was used (Radiation, 2000). The annual geometric mean concentration of ^222^Rn (Bq/l) was multiplied by the coefficient 2.8×10^-3^, and the effective dose received annually through inhaling ^222^Rn was estimated in mSv/y.

### 2.6 Statistical Analyses

Statistical analyses were done via SPSS 16, using One-way ANOVA method and correlation coefficient. The results were also stated in mean and standard deviation forms.

## 3. Results

Geometric mean and range of concentration of ^222^Rn in drinking water was measured 0.78±0.06 Bq/l and 0.19-1.7 Bq/l at 5 ºC and 0.46±0.04 Bq/l and 0.16-1.45 Bq/l at 15 ºC, respectively (p value<0.05). (Tables 1 and 2).

**Table 1:** Geometric mean (GM±SE), Middle, maximum and minimum concentration of ^222^Rn tap water samples in the temperature of 5 ºC (Bq/l) (n=250; Note 1)

Regions	Minimum	Maximum	Middle	Geometric mean
1	0.96	1.8	0.24	1.17±0.1
2	0.77	1.71	0.5	1.14±0.1
3	0.68	1.08	0.98	0.93±0.08
4	0.78	1.15	0.9	0.92±0.08
5	0.48	0.96	0.87	0.77±0.06
6	0.65	0.89	0.76	0.72±0.06
7	0.42	0.69	0.56	0.57±0.5
8	0.43	0.85	0.72	0.65±0.5
9	0.26	0.65	0.46	0.47±0.4
10	0.2	0.65	0.54	0.49±0.4

**Table 2:** Geometric mean (GM±SE), Middle, maximum and minimum concentration of ^222^Rn drinking water samples in the temperature of 15ºC (Bq/l) (n=250)

Regions	Min	Max	Middle	Geometric mean
1	0.6	1.14	0.78	0.81±0.6
2	0.54	1.45	0.76	0.86±0.7
3	0.26	0.82	0.59	0.53±0.4
4	0.48	0.88	0.62	0.62±0.5
5	0.17	0.75	0.53	0.48±0.4
6	0.19	0.75	0.47	0.43±0.3
7	0.18	0.49	0.32	0.33±0.2
8	0.16	0.49	0.39	0.37±0.3
9	0.17	0.54	0.29	0.27±0.2
10	0.2	0.42	0.26	0.27±0.2

**Figure 4:**
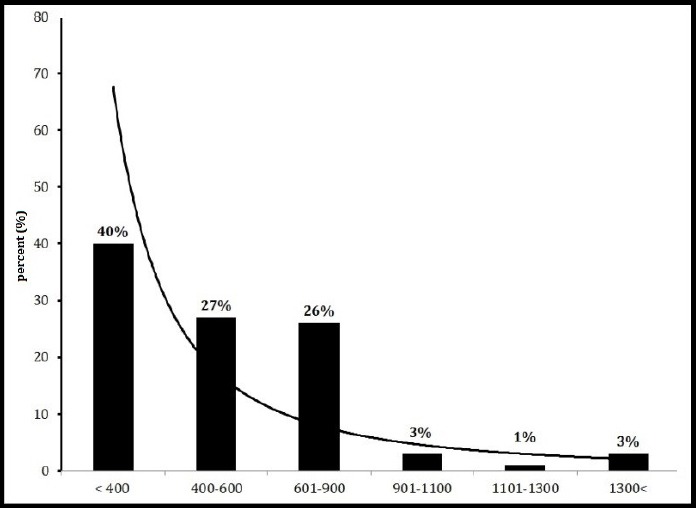
Percent of frequency distribution concentration of ^222^Rn in drinking water at 15 ºC

The percent of concentration frequency distributions of ^222^Rn in drinking water of 10 regions of Minab city in temperatures 5 and 15 ºC are shown in Figures 3 and 4. The maximum and minimum frequency distribution concentration of ^222^Rn at the temperature of 5 °C was observed in the range of 0.6-0.9 Bq/l and >0.4 Bq/l, respectively. At the temperature of 15 °C, they were observed in the range of >0.4 Bq/l and 1.1-1.3 Bq/l.

**Figurer 3:**
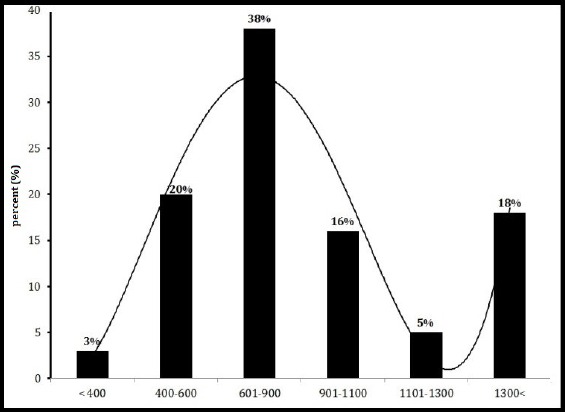
Percent of frequency distributions concentration of ^222^Rn Drinking water temperature in 5 ºC

The effective dose received annually through drinking water at the temperature of 5 °C in the children and adult groups was 0.011 and 0.007 mSv/y, respectively. At the temperature of 15 °C it was 0.007 and 0.003 mSv/y (p value<0.05). The effective dose received annually through inhaling ^222^Rn in drinking water at the temperatures of 5 °C and 15 °C were 0.0021 and 0.0012 mSv/y, respectively (p value<0.05).

**Figure 5:**
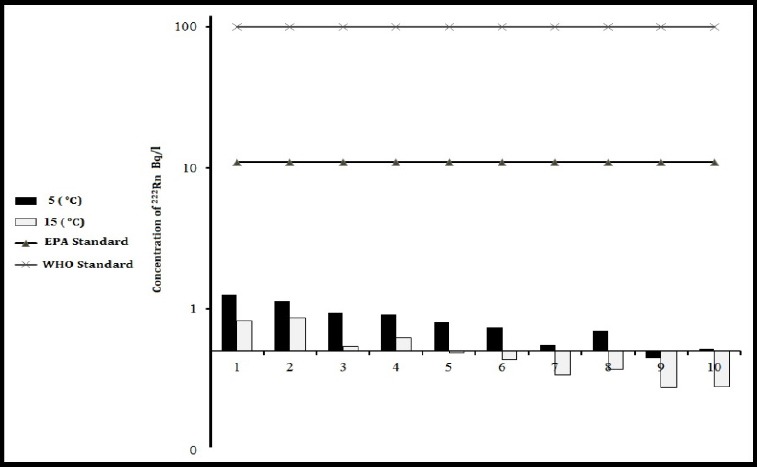
Geometric mean concentration of ^222^Rn drinking water in 10 regions of Minab at the temperatures of 5 °C and 15 °C

## 4. Discussion

Geometric mean concentration of ^222^Rn of drinking water at 5 **º**C (0.78±0.06 Bq/l) is greater than of 15 ºC (0.46±0.04 Bq/l). P value<0.05 between concentration of ^222^Rn of drinking water in temperatures 5 and 15 **º**C, indicate a significant difference. Consistent with our results, several studies reduce emissions ^222^Rn where the effect of reduced water solubility was observed as the temperature increased (Yalcin, Gurler, Akar, Incirci, & Kaynak, 2011; GalipYucea & Gasparonb, 2013; Oner, Yalim, Akkurt, & Orbay, 2009).

As it can be seen in Table 3, the range concentration of ^222^Rn in drinking water of Amasiya (0.39-1.17 Bq/l) is within the range concentration of ^222^Rn drinking water of Minab (0.16-1.7 Bq/l). The range concentration of ^222^Rn in drinking water of Tehran (27.7-74.3 Bq/l), Buvaji (12-41 Bq/l), Uberiya (5.9-65.7 Bq/l), and Islamabad (25.9-158.4 Bq/l) are much greater than Minab and Amasiya cities. Difference concentration of ^222^Rn in these towns (Amasiyia and Minab) may be due to different factors such as concentration of ^222^Rn in the water source, geological substrate type, water retention time and temperature during the measurement (Yiğitoğlu, Öner, Yalim, Akkurt, Okur, 2010). Groundwater resources (springs, wells, etc.) due to contact with the various layers of the earth, has more Total Dissolved Solid (TDS), including radioactive materials relative to surface waters (rivers, lakes, etc.). Layers of earth containing igneous rocks (granite) are larger, with higher concentration of radioactive material ^235^U (Rožmarić, Rogi, Benedik, & Štrok, 2012; Rezaei & Jalili-Majareshin, 2011). Since the ^222^Rn is the product of series of ^235^U decay, it can be expected that concentration in groundwater which cross from substrate type, are higher (Przylibski, Mamont-Cies´la, Kusyk, Dorda, & Kozlowska, 2004). Drinking water sources in Tehran, Buage, Umbria and Islamabad are of underground type similar to Minab and Amasia. However, concentration of 222Rn in the drinking water of Minab and Amasia is different. This could be due to differences in geological structure, measuring temperature and retention time of water. The effective dose received by children age group at the temperature of 5 °C (0.011 mSv/y) was 1.57 times more than of that at 15 °C (0.006 mSv/y). For adults it was 2 times bigger. Since the mean concentration of ^222^Rn at 5 °C (0.78±.06 Bq/l) is more than that of 15 °C (0.46±.04 Bql^-1^), the effective dose received is higher at this temperature. The effective dose received annually by adults and children from drinking water at 5 and 15 °C was below the standard 0.1 mSv/y. The activated coefficient of converting ^222^Rn to effective dose is higher in children group than adults (Somlai at al., 2007). The effective dose received from drinking water in this age group at the temperatures of 5 and 15 °C are 1.83 and 2.33 times bigger than adults’.

**Table 3:** Range concentration of ^222^Rn in tap water of Minab city compared with some other cities

Country / City	Water source	Range	References
Pakistan/Islamabad	Groundwater	25.9-158.4	(Ali, Khan, Akhter, Khan and Waheed, 2010)
Italy/Umberia	Groundwater	5.9-65.7	(Borio, Rongoni, Saetta, Desideri and Roselli, 2005)
Turkey/Amasiya	Groundwater	0.39-1.17	(Oner, Yalim, Akkurt and Orbay, 2009)
China/Bovaji	Groundwater	12-41	(Xinwei, 2006)
Iran/Tehran	Groundwater	27.7-74.3	(N.Alirezazadeh, 2005)
Iran/Minab	Groundwater	0.16-1.7	This Study

Effective dose due to inhalation of ^222^Rn from drinking water is much lower than the standard 1mSv/y effective dose due to ^222^Rn inhalation (Villalba, Sujoa, Cabrera, Jime´neza, & Villalobos, 2005). Effective dose received from ^222^Rn inhalation from drinking water at 5 ºC, is 1.82 times bigger than that of temperature 15 ºC. As can be seen in Table 4, the effective dose received by children (0.011 and 0.007 mSv/y) and adults (0.006 and 0.003 mSv/y) at a temperature 5 and 15 ºC of drinking water of Minab city is greater than Tehran (0.000129 and 0.00066 mSv/y), Balaton (0.0004 and 0.0002 mSv/y), Mashhad (0.00029 mSv/y for adult), Australia (0.005 mSv/y for adult), Gotaya (0.000122-0.0003 mSv/y) and Kastomono (0.00032-0.00093 mSv/y in summer and 0.00049-0.0008 mSv/y in the spring). ^222^Rn concentration range in drinking water of Tehran (27.7-74.3 Bq/l) is more than Minab City (0.16-1.7 Bq/l). However, due to the low effective dose conversion factor activity (0.35×10^-8^Sv/Bq) and capital annual consumption of water (children 75lit and adults 100lit), a lower effective dose is received by people of Tehran (N. Alirezazadeh, 2005). Lower effective dose in other cities can be due to low concentration of ^222^Rn, conversion factors and capital water consumption. Effective dose of induced inhalation in Minab (0.0021 and 0.0012 mSv/y) is higher than cities of Mashhad (0.0004 mSv/y), Gotiya (0.00003-0.00014 mSv/y) and is lower than cities of Tehran (0.01mSv/y), and Bovaji (0.03-0.14 mSv/y). However, effective dose of inhalation activity conversion factor of Minab (2.8 µSv/y) is higher than Tehran cities and Bovaji (1.8 µSvy^-1^) (Xinwei, 2006; N. Alirezazadeh, 2005), but due to the higher concentration of ^222^Rn in tap water, inhalation effective dose is higher in Minab city.

**Table 4:** Annual effective dose received by age groups of children and adults caused by the inhalation ^222^Rn and ingestion of tap water Minab (Iran) and other cities

City / Country	Annual effective dose			
	Drinking water (stomach) mSv/y		Inhalation (lung) mSv/y	References

	Childs	adults		
Minab/Iran^1^	0.011	0.006	0.0021	This study
Minab/Iran^2^	0.007	0.003	0.0012	This study
Mashhad/Iran	-	0.00029	0.0004	(Binesh, Mohammadi, Mowlavi, & Parvaresh, 2010)
Tehran/Iran	0.000129	0.00066	0.01	(N. Alirezazadeh, 2005)
Bovaji/China			0.03-0.14	(Xinwei, 2006)
Balaton/Netherlands	0.0004	0.0002		(Somlai at al., 2007)
Australia		0.005		(Kralik, Friedrich and Vojir, 2003)
Gotaya/Turkey	-	0.000122-0.0003	0.00014-0.00003	(Sahin, Çetinkaya, Saç, & Içhedef, 2013)
Kastomono/Turkey	-	0.00032-0.00093^3^ 0.00049-0.0008^4^	-	(Yalcin at al., 2011)

1 5 ºC Temperatures water;

2 15 ºC Temperatures water;

3 Summer season;

4 Spring season.

## 5. Conclusion

Geometric mean concentration of ^222^Rn in drinking water at temperatures 5 and 15 ºC (0.78±0.06 and 0.46±0.04 Bq/l) are lower than EPA and WHO standard limits. Annual Effective dose received from inhalation and consumption of ^222^Rn in drinking water in children is more than adults (p value<0.05). Also, effective dose received in both age groups, are much lower than EPA and WHO standard limits. Increasing the temperature reduces the effect on concentration of ^222^Rn in drinking water, followed by a reduction in received effective dose. Hence, it is recommended to reduce the effective dose received in the cities with high concentration of ^222^Rn in drinking water, water ingestion be at higher temperature.
